# Cytoplasmic Cyclin E Is an Independent Marker of Aggressive Tumor Biology and Breast Cancer-Specific Mortality in Women over 70 Years of Age

**DOI:** 10.3390/cancers12030712

**Published:** 2020-03-18

**Authors:** Simon J. Johnston, Binafsha M. Syed, Ruth M. Parks, Cíntia J. Monteiro, Joseph A. Caruso, Andrew R. Green, Ian O. Ellis, Kelly K. Hunt, Cansu Karakas, Khandan Keyomarsi, Kwok-Leung Cheung

**Affiliations:** 1 Nottingham Breast Cancer Research Centre, School of Medicine, The University of Nottingham, Nottingham NG7 2RD, UK; simon.johnston@cantab.net (S.J.J.); binafsha.syed@lumhs.edu.pk (B.M.S.); ruth.parks@nottingham.ac.uk (R.M.P.); cintiamonteiro@yahoo.com.br (C.J.M.); andrew.green@nottingham.ac.uk (A.R.G.); ian.ellis@nottingham.ac.uk (I.O.E.); 2 Gene Regulation Laboratory, School of Pharmacy, The University of Nottingham, Nottingham NG7 2RD, UK; 3 Medical Research Centre, Liaquat University of Medical and Health Sciences, Jamshoro 76080, Pakistan; 4 Department of Pathology, University of California, San Francisco, CA 94143, USA; joseph.caruso2@ucsf.edu; 5 Department of Breast Surgical Oncology, The University of Texas MD Anderson Cancer Center, Houston, TX 77030, USA; khunt@mdanderson.org; 6 Department of Experimental Radiation Oncology, The University of Texas MD Anderson Cancer Center, Houston, TX 77030, USA; cansu_karakas@yahoo.com (C.K.); kkeyomar@mdanderson.org (K.K.)

**Keywords:** breast cancer, cyclin E, older patients, biomarker, tumor biology, prognosis, survival

## Abstract

Multi-cohort analysis demonstrated that cytoplasmic cyclin E expression in primary breast tumors predicts aggressive disease. However, compared to their younger counterparts, older patients have favorable tumor biology and are less likely to die of breast cancer. Biomarkers therefore require interpretation in this specific context. Here, we assess data on cytoplasmic cyclin E from a UK cohort of older women alongside a panel of >20 biomarkers. Between 1973 and 2010, 813 women ≥70 years of age underwent initial surgery for early breast cancer, from which a tissue microarray was constructed (*n* = 517). Biomarker expression was assessed by immunohistochemistry. Multivariate analysis of breast cancer-specific survival was performed using Cox’s proportional hazards. We found that cytoplasmic cyclin E was the only biological factor independently predictive of breast cancer-specific survival in this cohort of older women (hazard ratio (HR) = 6.23, 95% confidence interval (CI) = 1.93–20.14; *p* = 0.002). At ten years, 42% of older patients with cytoplasmic cyclin E-positive tumors had died of breast cancer versus 8% of negative cases (*p* < 0.0005). We conclude that cytoplasmic cyclin E is an exquisite marker of aggressive tumor biology in older women. Patients with cytoplasmic cyclin E-negative tumors are unlikely to die of breast cancer. These data have the potential to influence treatment strategy in older patients.

## 1. Introduction

The prognosis for patients with primary breast cancer is a function of disease extent at presentation and tumor biology. Time-dependent factors, including metastatic involvement of draining axillary lymph nodes (stage) and the size of the primary tumor, indicate disease extent, while the degree of differentiation (grade) is used as a surrogate for biological aggressiveness. These histopathology-assessed features are classically combined into the Nottingham Prognostic Index (NPI) [1].

Due to comorbidities, older patients with primary breast cancer are more likely to die of non-breast cancer-related causes when compared to their younger counterparts [2,3]. Furthermore, tumors in older patients have distinct biological characteristics that are linked to favorable outcome, such as a higher proportion and degree of hormone receptor (estrogen receptor (ER) and/or progesterone receptor (PR)) positivity, and low histological grade and Ki67 proliferative index [4,5]. Biomarkers that may influence treatment strategy therefore require interpretation in the specific clinical and biological context of older women.

Recent developments in risk stratification, such as tumor gene expression profiling (e.g., Oncotype Dx and MammaPrint), have focused on the biology of the primary tumor, based on the premise that this reflects the biology of presumed micrometastatic deposits [6,7]. Adjuvant therapy is then selected according to risk category with the aim of preventing these deposits from developing into clinically detectable metastases, thus avoiding disease relapse.

We have previously shown that tumor biology in older (>70 years) versus younger primary breast cancer cohorts can be distinguished by immunohistochemistry (IHC) using a panel of biomarkers, including ER, PR, HER2, Ki67, Bcl2, BRCA1, BRCA2, E-Cadherin, EGFR, LKB1, MDM2, MDM4, MUC1, p53, VEGF and cytokeratin markers of luminal and basal disease [4]. Differential expression of age-associated biomarkers clustered tumors from older patients into six groups with distinct clinical outcomes.

The current study focuses on the role of G1/S-specific cyclin-E1 (cyclin E) as a biological marker of aggressive disease, and on its prognostic significance in the context of older women with primary breast cancer.

In normal dividing cells, cyclin E promotes the transition from G1 to S phase by activating cyclin-dependent kinase 2 (CDK2) [8]. In breast cancer cells, tumor-specific proteolytic processing of cyclin E generates hyperactive low molecular weight (LMW) isoforms [9]. In contrast to full length cyclin E, for which prognostic data have been equivocal, LMW isoforms are highly prognostic in primary breast cancer patients [10,11,12]. As they lack a portion of the amino-terminus containing a nuclear localization sequence, LMW isoforms of cyclin E preferentially accumulate in the cytoplasm where they evade nuclear FBW-7 ubiquitin ligase that would otherwise increase their turnover [13].

**Figure 1:**
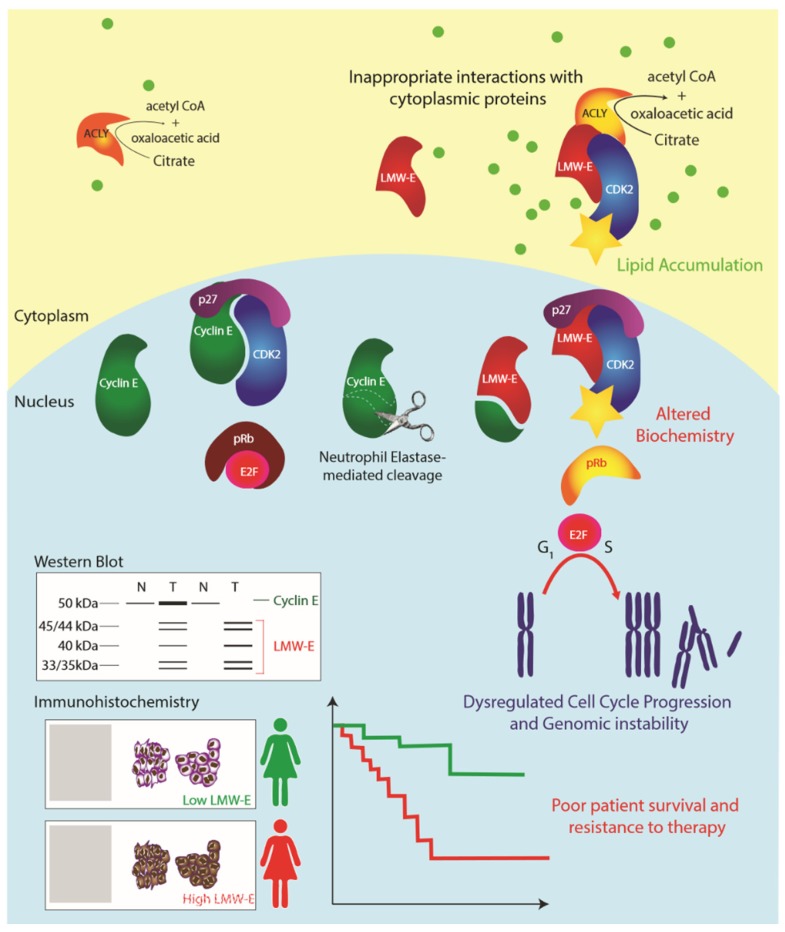
Model: Low molecular weight cyclin E (LMW-E) isoforms are generated by neutrophil elastase-mediated proteolytic cleavage, removing the N-terminal nuclear localization signal. LMW-E isoforms accumulate in the cytoplasm where they inappropriately interact with cytoplasmic proteins, such as ACLY. The altered biochemistry of LMW-E results in hyperactivation of CDK2, resistance to endogenous CDK-inhibitors (p21CIP1 and p27KIP1) and altered substrate interactions, which results in enhanced cell cycle progression, genomic instability and other pro-tumorigenic features. Evaluation of LMW-E by Western blot (protein size) or immunohistochemistry (cytoplasmic localization) is prognostic of poor prognosis and predicts failure of standard treatment modalities.

In previous work using an antibody named C-19 that interacts with the carboxyl-terminus of cyclin E (present in both full length and LMW isoforms of cyclin E), we showed that transgenic mouse models with mammary gland expression of LMW cyclin E developed mammary tumors positive only for cytoplasmic cyclin E [14]. Furthermore, cytoplasmic cyclin E bound to its catalytic subunit, CDK2, in the cytoplasm of tumor cells [14]. The biological functions of cytoplasmic, LMW cyclin E are summarized in Figure 1.

These critical findings on the biological functions of LMW cyclin E paved the way for a pivotal study on the use of cytoplasmic expression of cyclin E (c-cyclin E) by IHC to predict recurrence in patients with primary breast cancer [15]. Combined analysis of 2494 tumors from four cohorts of patients (from the UK and the USA, covering all age groups) presenting with primary breast cancer demonstrated that c-cyclin E staining is highly prognostic. These data included the current UK cohort of older women (median age 76, versus 53–62 years in all cohorts, *p* < 0.0001).

We now present data on c-cyclin E exclusively from the Nottingham cohort. This paper is distinct from the multi-cohort study as it focuses only on the older population and assesses the role of c-cyclin E against a large panel of more than 20 disease markers. Findings are interpreted in the specific biological and clinical context of primary breast cancer in older women, and the implications for risk stratification and treatment decision-making in older patients are discussed.

## 2. Results

Patient clinicopathological characteristics are summarized in Table 1. Median follow-up was 6.3 years (95% CI, 6.1–7.1 years).

### 2.1. Expression of Cytoplasmic Cyclin E Associates with High-Grade, ER-/PR- Breast Tumors with High Ki67 Proliferative Index

We examined the expression of c-cyclin E and a panel of biomarkers in breast tumors by IHC. Average concordance between pathologists was 93% for the Nottingham cohort (see Table 2).

**Figure 2:**
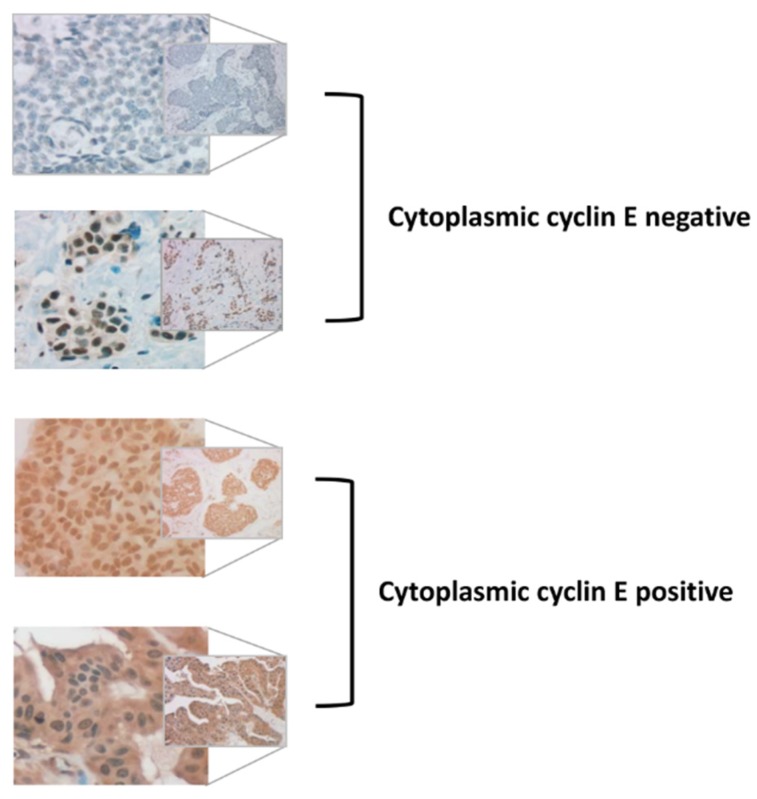
Representative images of positive and negative staining for cytoplasmic cyclin E using antibody C-19.

Representative IHC staining for c-cyclin E positive and negative cases is presented in Figure 2.

To assess the potential clinical relevance of c-cyclin E, we first compared c-cyclin E expression with clinicopathological features at initial diagnosis (age, tumor size, stage and grade) and markers of disease biology in current clinical use, including ER, PR, HER2 and Ki67 status.

There was a strong association between c-cyclin E expression and higher grade (*p* < 0.0005, see Table 3). In contrast, there was no association between c-cyclin E and patient age, tumor size or stage. Cytoplasmic expression of cyclin E was significantly associated with negative ER and PR status (*p* = 0.002 and *p* = 0.012, respectively) and high Ki67 proliferative index (*p* = 0.047) (Table 3). No significant association was found between c-cyclin E and HER2 status.

Comparison of c-cyclin E status with other biomarkers revealed a positive association with VEGF (*p* = 0.041), and no other significant association.

### 2.2. Cytoplasmic Cyclin E Expression Is Enriched in Basal Tumors

We next assessed the association between tumor c-cyclin E expression and cellular phenotype as indicated by the expression of cytokeratin markers in the IHC protein panel.

Cytoplasmic cyclin E expression was associated with markers of basal disease (see Table 4). Basal cytokeratin markers significantly associated with c-cyclin E included CK5 and CK17 (*p* = 0.001 and *p* = 0.036, respectively). In contrast, there was no association between c-cyclin E and the luminal marker CK18.

### 2.3. Survival Analysis

**Figure 3:**
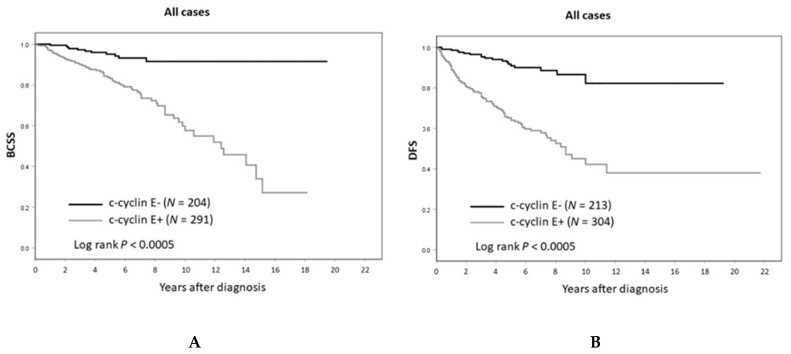
(**A**) Breast cancer-specific and (**B**) disease-free survival by cytoplasmic cyclin E status.

**Figure 4:**
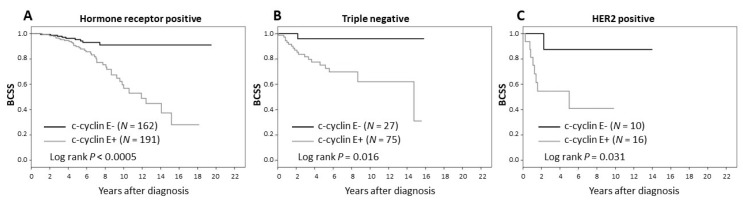
Breast cancer-specific survival by subtype: (**A**) hormone receptor (ER and/or PR) positive, (**B**) triple negative, (**C**) HER2 positive.

Kaplan–Meier plots of breast cancer-specific survival (BCSS) and disease-free survival (DFS) as a function of c-cyclin E status are shown in Figure 3. Lack of c-cyclin E was associated with good prognosis in the patient cohort (BCSS and DFS both *p* < 0.0005 by logrank test). This was observed for luminal A/B (ER+ and/or PR+), HER2+ and triple negative breast cancer subtypes (see Figure 4).

Survival analysis of c-cyclin E alongside the full panel of biomarkers was performed using data up to last follow-up. Due to the low proportion of low-grade tumors (grade 1, 12%), these were combined with intermediate-grade tumors (grade 2, 40%) and used as a statistical reference for comparison with high-grade tumors (grade 3, 48%). Multivariate analysis was performed on all clinicopathological factors and biomarkers significantly associated with BCSS in univariate testing.

**Figure 5:**
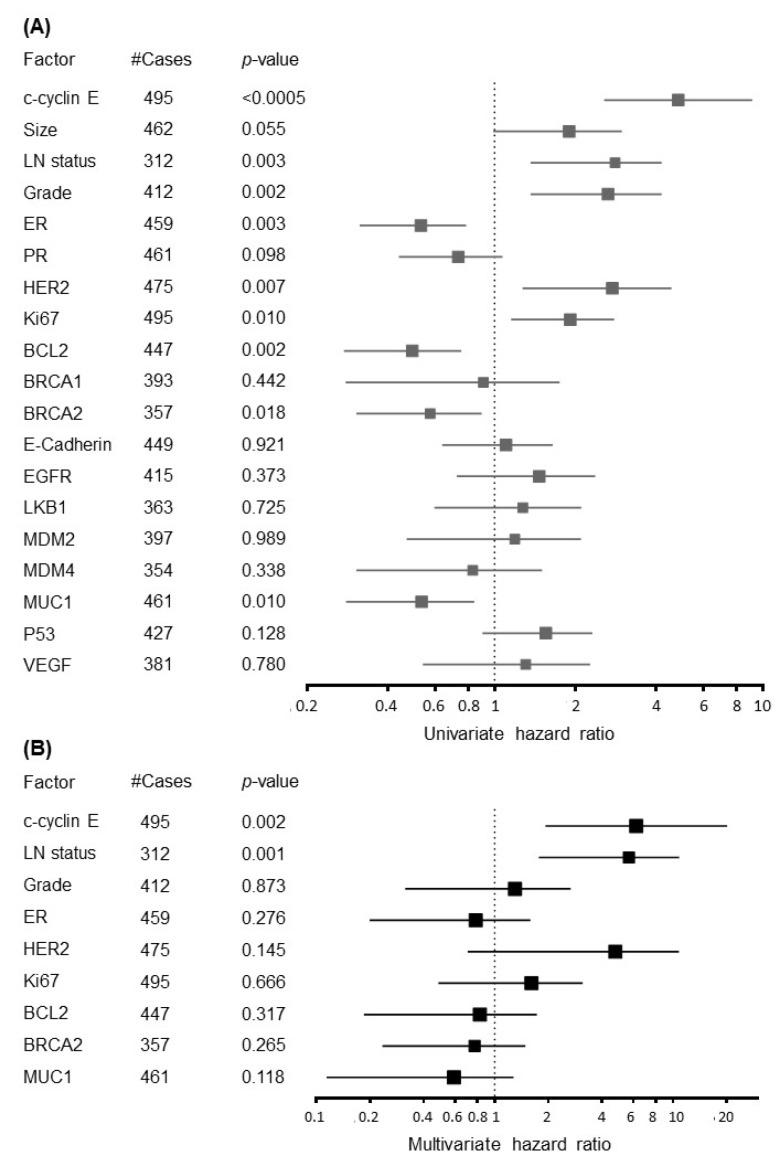
(**A**) Univariate and (**B**) multivariate analysis of c-cyclin E with clinicopathological and age-associated biomarkers.

Cytoplasmic expression of cyclin E was the only independent biomarker of BCSS and had a strong association in multivariate analysis (HR = 6.23, 95% CI 1.93–20.14; *p* = 0.002) (Figure 5). The only clinicopathological factor predictive of BCSS in the multivariate analysis was axillary nodal status (HR = 4.38, 95% CI 1.77–10.84; *p* = 0.001).

For the whole cohort of 517 patients, there was a strong positive association between c-cyclin E positivity and breast cancer-specific mortality at 5 years of follow-up (*p*<0.0005). Multivariate analysis demonstrates that c-cyclin E positivity is the strongest predictor of 5-year BCSS in this cohort (HR = 9.12, 95% CI 2.22–37.40; *p* = 0.002)—outperforming lymph node status (HR=4.49, 95% CI 1.66–12.15; *p* = 0.003) and all other biological disease markers.

**Figure 6:**
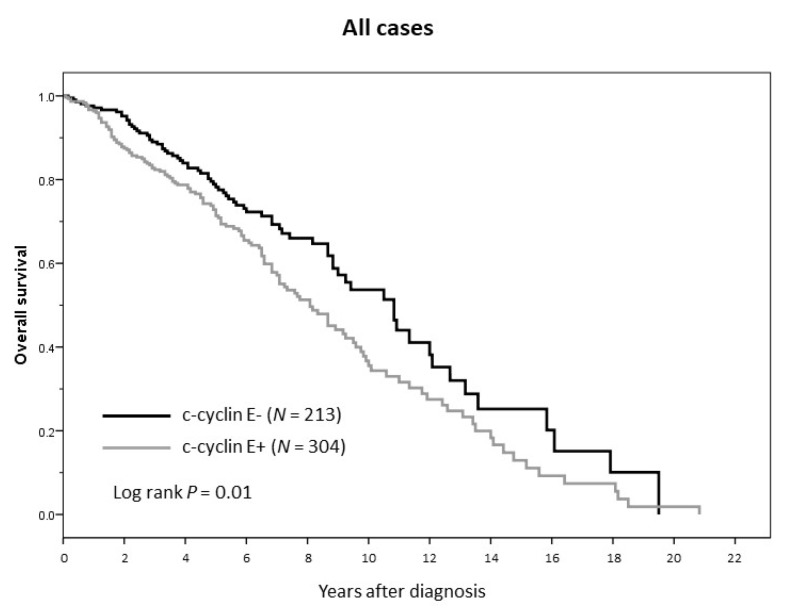
Overall survival (all cases) by cytoplasmic cyclin E status.

At ten years of follow-up, BCSS for patients with c-cyclin E-negative tumors was 92% versus 58% for those with c-cyclin E-positive tumors (HR = 6.23, 95% CI 1.92–20.14; *p* = 0.002 in multivariate analysis). At completion of follow-up, the absolute difference in median overall survival (OS) by c-cyclin E status (positive versus negative) was 33 months (130 versus 97 months, *p* = 0.01 by logrank, Mantel–Cox) (see Figure 6).

### 2.4. Prognostic Value of Cytoplasmic Cyclin E in Patients Treated with Endocrine Therapy

Patients with ER+ disease received up to 5 years of adjuvant endocrine therapy according to risk stratification by NPI (e.g., NPI > 2.4 or 3.4, according to best practice guidelines at the time of treatment). We next explored the prognostic value of c-cyclin E and its relationship to adjuvant endocrine therapy in patients with ER+ tumors.

**Figure 7:**
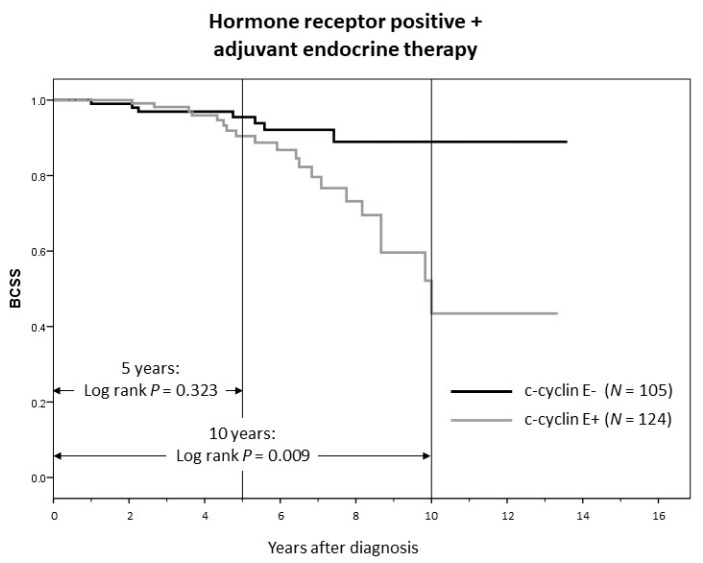
Adjuvant endocrine therapy negates the poor prognostic effect of c-cyclin E positivity, but only for as long as it is given (up to 5 years).

In contrast to the whole cohort (where c-cyclin E was the strongest predictor of 5-year BCSS), in patients who received up to 5 years of adjuvant endocrine therapy (*n* = 229), c-cyclin E was not associated with 5-year BCSS (*p* = 0.323). After cessation of adjuvant endocrine therapy, c-cyclin E reached prognostic significance (positive association with breast cancer-specific mortality) at 10 years of follow-up (*p* = 0.009) (see Figure 7).

## 3. Discussion

In this study of 517 older women with primary breast cancer, c-cyclin E is the only independent biological marker of disease outcome. In terms of prognostic value, c-cyclin E status outperforms all standard clinical and age-associated biomarkers tested in this study.

For the older population, who more often present with complex comorbidities and psychosocial factors, considerations regarding competing causes of death are paramount in clinical decision-making [16]. The current study presents BCSS as a surrogate measure for the impact of tumor biology on patient mortality. Deaths due to non-breast cancer causes are excluded from the analysis. Unlike non-modifiable and time-dependent clinicopathological disease factors, such as tumor size and stage, those factors pertaining to tumor biology can be modified using systemic therapy.

Data from the current study supplement findings from an international study of four cohorts (including the Nottingham cohort) and add clinical context in older women [16]. In the combined analysis, freedom from recurrence (FFR), defined as the time between the date of diagnosis and the date of first locoregional or distant recurrence, was the primary endpoint. Unlike BCSS and DFS, FFR does not include death as an event, regardless of the cause of death.

In two of the other three cohorts in the combined analysis (MD Anderson (MDA) and National Cancer Institute (NCI)), c-cyclin E expression had the strongest effect on FFR. In the Nottingham cohort, c-cyclin E outperformed standard clinical biomarkers in terms of both FFR and BCSS.

Combined analysis of the two largest US clinical cohorts contributing to the multi-cohort study has also been reported (MDA and NCI) [14]. Data from the Nottingham cohort are consistent with the NCI and MDA cohorts. For example, there was 60% positive c-cyclin E staining in combined NCI/MDA analysis, compared to 59% in the Nottingham cohort. This demonstrates good reproducibility of IHC measurement of cytoplasmic expression of cyclin E—an important factor when considering the potential application of c-cyclin E status as a clinical biomarker.

In line with the Nottingham cohort, expression of c-cyclin E in the combined analysis of the NCI and MDA cohorts was associated with tumor markers, such as grade, ER and PR, and was associated with poor patient outcome. However, in the NCI/MDA analysis, c-cyclin E was only an independent marker of recurrence-free and overall survival when combined with the downstream kinase, cyclin-dependent kinase 2. In the NCI/MDA analysis, histological grade was also an independent prognostic factor. 

Analysis of the Nottingham cohort of older women, in contrast, found that c-cyclin E was the only independent prognostic biomarker, outperforming even tumor grade in terms of prognostic value. Although c-cyclin E was not associated with age in any of the cohorts, overall findings from the older Nottingham cohort suggest a greater biological effect of high c-cyclin E expression in the older population. Breast cancer biology in the older population is considered more indolent, with less aggressive phenotypic features and enrichment for ER expression. This suggests that c-cyclin E has greater potential clinical utility in the older population and is better able to distinguish more aggressive tumors that are likely to lead to death from breast cancer.

Findings from the current study support c-cyclin E as a robust and reproducible biomarker of an aggressive disease course. The findings suggest that clinical outcomes of older women with primary breast cancer can be predicted from the biology of their tumor using c-cyclin E alone. Older patients with c-cyclin E-negative tumors are unlikely to die of their breast cancer. At ten years, only 8 patients out of 100 will die of their breast cancer if their tumor is c-cyclin E-negative. For many older patients, this finding will directly impact clinical decision-making.

Alongside existing clinical decision-making tools such as comprehensive geriatric assessment scales and biomarkers of treatment response such as ER expression, there is clear potential to utilize tumor c-cyclin E expression in the clinic. For example, c-cyclin E positivity may indicate a requirement for an aggressive initial treatment strategy, in terms of surgical management of the primary tumor and axillary lymph nodes, and the need for adjuvant therapy.

Although the clinical setting of the current study is patients selected to undergo initial surgery, who are more likely to have ER-negative tumors, it may be possible to extrapolate its findings on the value of c-cyclin E as a marker of aggressive disease biology to other clinical scenarios. 

For example, ER/PR+ primary tumors that do not express c-cyclin E may be adequately treated by primary endocrine therapy. Alongside comprehensive geriatric assessment, c-cyclin E may serve as a biomarker in this context for patients who, because of comorbidity, psychosocial factors or individual choice, would prefer not to undergo initial surgery. Given the high proportion of older patients already receiving primary endocrine therapy (up to 40% of UK patients in the previous decade), there is a compelling case to investigate this hypothesis in prospective clinical trials [17].

In adjuvant therapy decision-making, c-cyclin E could supplement or replace gene expression predictors of chemotherapy response (e.g., Oncotype Dx and MammaPrint). Additionally, as cyclin-dependent kinase 2-targeting treatments, e.g., dinaciclib, emerge from early phase clinical trials, there may be a role for these drugs in older patients in preference to chemotherapy [18,19].

It is reported that c-cyclin E mediates resistance to endocrine therapy with aromatase inhibitors (AIs) in breast cancer [20]. This study suggests that c-cyclin E may identify ER/PR+ tumors that are unresponsive to AIs, which do not induce a cytostatic effect.

For patients with ER+ disease, evidence from the current study suggests that adjuvant endocrine therapy negates the poor prognostic effect of c-cyclin E positivity. This implies that extending adjuvant therapy beyond 5 years would improve long-term outcomes and would be a priority for patients with c-cyclin E-positive tumors.

## 4. Materials and Methods

Over a 37-year period (1973–2010), 1758 older (≥70 years) women with stage I–II primary breast cancer were managed in a dedicated facility in Nottingham, as previously described [21]. Of these patients, 813 underwent primary surgery.

Good quality formalin-fixed, paraffin embedded tissue from 575 tumors was available, from which 517 were successfully incorporated into a tissue microarray (TMA), as previously described [22]. Briefly, representative 0.6mm diameter cores were implanted in the TMA block using Beecher’s manual tissue microarray (MP06 Beecher Instruments Inc., Sun Prairie, WI, USA).

Clinical information was available from diagnosis to death or last follow-up. Patient clinicopathological characteristics are summarized in Table 1. Median follow-up was 6.3 years (95% CI, 6.1–7.1 years).

The TMA was used to test expression of a panel of biomarkers by IHC, using the StreptAvidin Biotin Complex and EnVision methods, which have been extensively described [4]. Biomarkers tested in Nottingham included ER, PR, HER2, Ki67, Bcl2, BRCA1, BRCA2, E-Cadherin, EGFR, LKB1, MDM2, MDM4, MUC1, p53, VEGF and cytokeratin markers CK5, CK5/6, CK14, CK17 and CK18. 

Staining of the biomarker panel was assessed as previously reported [4]. Cytoplasmic staining for cyclin E was centrally assessed at MD Anderson Cancer Center as previously described, using Santa Cruz clone C-19 polyclonal antibody to cyclin E [15]. TMA results were interpreted by two independent pathologists blinded to clinical outcome and assigned according to percentage of cells stained and intensity of staining. Cut-off values for all biomarkers were as reported in our previous studies [4,14].

Conventional pathological parameters as part of standard reporting for surgical specimens at Nottingham were measured, including size, grade and axillary stage (regional lymph node involvement). The policy of axillary surgery evolved according to clinical evidence-based guidelines over the 37-year period of sample collection, as previously described [21].

The primary endpoint was breast cancer-specific survival (BCSS), defined as time from diagnosis to last follow-up or death from breast cancer (i.e., excluding death due to competing causes). Secondary endpoints included disease-free survival (DFS), defined as time from diagnosis to first recurrence, and overall survival (OS), defined as time from diagnosis to last follow-up or death from any cause.

The bioinformatics software X-Tile was used to define thresholds for biomarker positivity, as previously described [23]. Biomarker expression was compared between groups by χ^2^ test. Kaplan–Meier survival analysis was performed using logrank and generalized Wilcoxon tests. Multivariate analysis of BCSS was performed using the Cox regression model. Statistical significance was defined as *p* < 0.05. Reporting adhered to reporting recommendations for tumor marker prognostic studies (REMARK) criteria [24].

This research was conducted in accordance with the Declaration of Helsinki. The study protocol was approved by the Institutional Review Boards of the University of Nottingham, Nottingham University Hospitals NHS Trust and the University of Texas MD Anderson Cancer Center (MDA ref.33).

## 5. Conclusions

This study highlights the clinical value of tumor c-cyclin E status as a strong predictor of the biological course of primary breast cancer in older patients. Those who present with c-cyclin E-negative tumors are unlikely to die of their breast cancer. Alongside existing tools such as geriatric assessment and biomarkers of treatment response such as ER positivity, c-cyclin E status may assist initial therapy decision-making in terms of intensity, duration and modality. These hypotheses warrant prospective clinical evaluation in the specific clinical context of primary breast cancer in older women.

## Figures and Tables

**Table 1:** Summary of patient characteristics (*n* = 517).

Variable	Group	*n*	%
**age**	70 to <75 yrs	206	39.8%
	75 to <80 yrs	202	39.1%
	80 to <85 yrs	83	16.1%
	≥85 yrs	26	5.0%
**tumor size**	<20 mm	153	31.7%
	≥20 mm	329	68.3%
**stage**	1	183	57.5%
	2	98	30.8%
	3	37	11.6%
**grade**	1	51	12.0%
	2	169	39.8%
	3	205	48.2%
**ER status**	negative	143	29.9%
	positive	335	70.1%
**HER2 status**	negative	453	91.7%
	positive	41	8.3%
**Metastasis ^1^**	negative	430	83.2%
	positive	87	16.8%

^1^ at last follow-up.

**Table 2:** Concordance between two independent pathologists for the Nottingham cohort (*n* = 516).

		Pathologist B		Total
		c-cyclin E+	c-cyclin E−	
**Pathologist A**	**c-cyclin E+**	199	11	210
	**c-cyclin E−**	11	295	306
				516

**Table 3:** Association between tumor c-cyclin E status and clinicopathological factors.

Variable		c-Cyclin E−		c-Cyclin E+		*p*
**age**	70 to <80 yrs	170	(41.7%)	238	(58.3%)	0.743
	≥80 yrs	43	(39.4%)	66	(60.6%)	
**size**	<20 mm	64	(41.8%)	89	(58.2%)	0.921
	≥20 mm	135	(41.0%)	194	(59.0%)	
**stage**	1	83	(45.4%)	100	(54.6%)	0.350
	2	42	(42.9%)	56	(57.1%)	
	3	12	(32.4%)	25	(67.6%)	
**grade**	1	33	(64.7%)	18	(35.3%)	<0.0005 ^1^
	2	87	(51.5%)	82	(48.5%)	
	3	64	(31.2%)	141	(68.8%)	
**ER**	negative	43	(30.1%)	100	(69.9%)	0.002 ^1^
	positive	151	(45.1%)	184	(54.9%)	
**PR**	negative	74	(34.6%)	140	(65.4%)	0.012 ^1^
	positive	122	(46.2%)	142	(53.8%)	
**HER2**	negative	187	(41.3%)	266	(58.7%)	0.869
	positive	16	(39.0%)	25	(61.0%)	
**Ki67**	negative	152	(44.3%)	191	(55.7%)	0.047 ^1^
	positive	61	(35.1%)	113	(64.9%)	

^1^ Statistical significance *p* < 0.05, by χ^2^ test

**Table 4:** Association between tumor c-cyclin E status and clinicopathological factors.

Variable		c-Cyclin E−		c-Cyclin E+		*p*
**CK5**	negative	152	(45.5%)	182	(54.5%)	0.001 ^1^
	positive	43	(29.5%)	103	(70.5%)	
**CK5/6**	negative	101	(43.3%)	132	(56.7%)	0.284
	positive	78	(37.9%)	128	(62.1%)	
**CK14**	negative	143	(43.7%)	184	(56.3%)	0.074
	positive	37	(33.9%)	72	(66.1%)	
**CK17**	negative	160	(43.1%)	211	(56.9%)	0.036 ^1^
	positive	30	(31.3%)	66	(68.8%)	
**CK18**	negative	3	(21.4%)	11	(78.6%)	0.170
	positive	187	(41.9%)	259	(58.1%)	

^1^ statistical significance *p* < 0.05, by χ^2^ test. CK5, CK5/6 (antibody to both CK5 and CK6), CK14 and CK17 are basal markers; CK18 is a luminal marker.
